# Supplementary figures and images for: Polymorphic Appetite Effects on Waist Circumference Depend on rs3749474 CLOCK Gene Variant
Source: Nutrients. 2020 Jun 21;12(6):1846. doi: 10.3390/nu12061846 (PMC7353411; doi:10.3390/nu12061846)

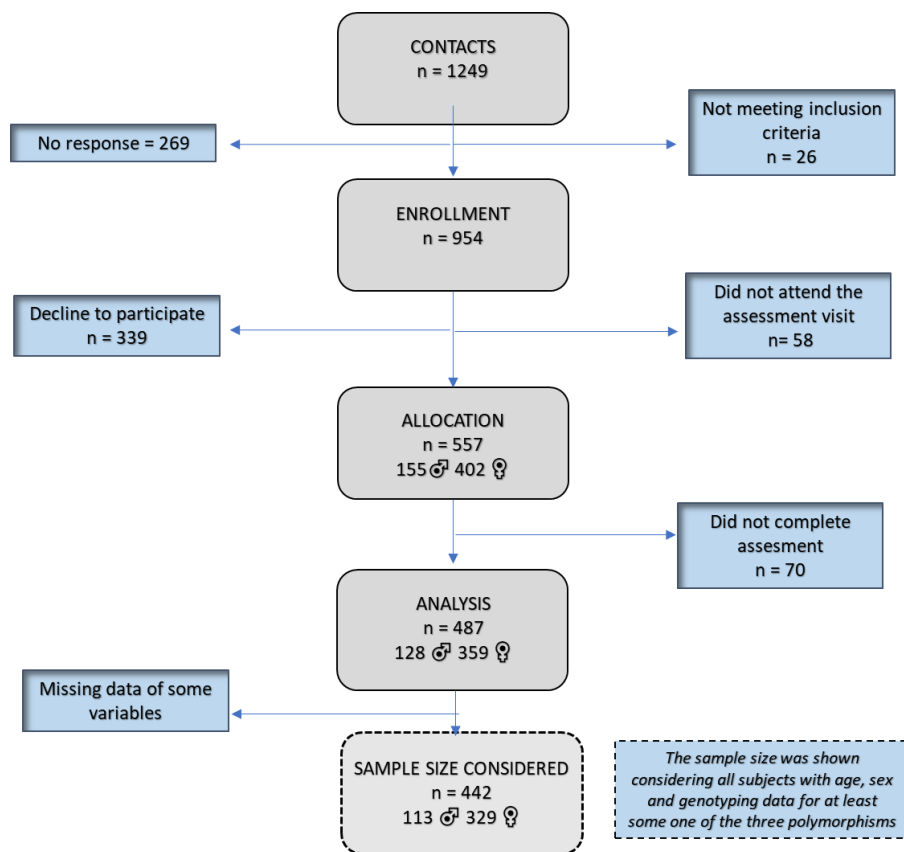

Figure S1. CONSORT flow diagram.

Supplement: Supplementary file 1 [file nutrients-12-01846-s001.pdf]
